# Hunger as an uncontroversial predictor of poor adolescent mental health: evidence from a multiverse analysis of 410,213 adolescents across 79 countries

**DOI:** 10.1111/jcpp.70176

**Published:** 2026-06-02

**Authors:** Mirela Zaneva, Tsvetomira Dumbalska, Aaron Reeves, Lucy Bowes

**Affiliations:** ^1^ Christ Church College University of Oxford Oxford UK; ^2^ Department of Experimental Psychology University of Oxford Oxford UK; ^3^ Department of Sociology London School of Economics and Political Science London UK

**Keywords:** Global mental health, hunger, food insecurity, multiverse analysis

## Abstract

**Background:**

Hunger has established detrimental impacts on physical health, with emerging evidence indicating negative impacts on mental health. However, there is a pronounced knowledge gap outside high‐income settings and for adolescents. Previous research also provides differing estimates of hunger's impacts, potentially underpinned by a wide range of researcher degrees of freedom.

**Methods:**

We investigated the relationship between hunger and mental health (proxied by single‐item indicator for worrying and three items for suicidality) in the Global School‐based Student Health Survey, combining datasets from 79 countries and in a pooled sample of 410,213 adolescents (mean age 14.72 ± 1.56 years; 52% girls). We used a principled multiverse analysis, exploring 15,360 model specifications.

**Results:**

Our results show a consistently stable negative relationship between hunger and adolescent mental health (worrying, median beta = .12; suicidality, median beta = .07). This reflects 12 and 7 percentage point increases in the *prevalence* for worry and suicidality, respectively, when experiencing hunger. This relationship is significant across a variety of analytical choices including sample selection, covariate choice (including country‐level controls for wealth and inequality), predictor and outcome manipulation, among others. In a further multiverse analysis, we find evidence of a dose–response relationship, such that more frequently experiencing hunger is associated with greater reported levels of worrying and suicidality.

**Conclusions:**

Our work attests to the experience of hunger as one of the most clearly uncontroversial predictors of poor mental health. Addressing food insecurity and the equitable and efficient distribution of food globally is a crucial consideration for adolescent mental health protection and suicide prevention. Emphasis should be placed on appropriate population‐level interventions, such as universal free school or community meal programs.

## Introduction

Hunger and food insecurity have profound negative impacts on societies worldwide. An estimated 2.8 billion people currently cannot afford a healthy diet and 733 million globally suffered from malnutrition in 2023 (FAO, IFAD, UNICEF, WFP and WHO, 2024). Hunger refers to individual level, typically subjectively reported experiences of going without food, which can be episodic or chronic, and expressed by physical sensations of discomfort, weakness, pain. Hunger can be a consequence or marker of food insecurity. Food insecurity refers to the limited or uncertain availability of safe and nutritionally adequate foods, which is typically measured at household or higher order (e.g. neighborhood, region) contextual levels.

We know that hunger harms health. Evidence from systematic reviews reveals deleterious impacts on anemia, diabetes, blood pressure, heart disease, asthma, and gum disease, among others (Moradi et al., 2019). Most of this research focuses on adults in high‐income countries and until recently largely ignored mental health, with some studies suggesting hunger negatively affects adults' anxiety and depression (Pourmotabbed et al., 2020). Indeed, a further systematic review found that only one‐third of studies on the relationship between food insecurity (including hunger) and mental health come from low‐ and middle‐income countries (LMICs), despite over 80% of the global population living in LMICs; further, over 70% of the included studies surveyed general adult or older adult populations (Sparling et al., 2022). In other words, we know comparatively less about the impacts of hunger on children and adolescents' mental health; we also cannot generalize findings from high‐income settings to LMICs (Campbell, Bann, & Patalay, 2021). Previous research has often focused on ‘easier’ to measure outcomes like nutrition or anthropometrics (like height and weight (Sparling et al., 2022; Weaver & Hadley, 2009)). However, subjective experiences of hunger can capture psychological and emotional impacts beyond biological needs. Moreover, they could better reflect lived experience, including personal preferences, social and economic context (e.g. social comparison, stigma). This knowledge gap is especially important given that early intervention can disrupt hunger's negative impacts, which include increased risk for depression and suicide throughout life (McIntyre, Williams, Lavorato, & Patten, 2013).

To understand the unique effects of hunger on young people's mental health, we need more diverse data both at the population and individual level to avoid misgeneralization (Patel, 2007). Significant challenges in studying the effects of hunger (and food insecurity and poverty more broadly), include the need for large‐scale global survey efforts, and use of validated and robust measures (Burns, 2015). Another challenge is the diversity of plausible analytical approaches. Existing research illustrates how the magnitude and even *statistical significance* of the effects can change with different analytical decisions. For instance, existing work illustrates that choices about the inclusion or exclusion of covariates (Melchior et al., 2012; Steare et al., 2024); the operationalization of hunger/food insecurity (e.g. categorizing severity with different cut‐offs (Weinreb et al., 2002)); and sample selection (e.g. region/country (Steare et al., 2024)) have produced nonsignificant associations with adolescent mental health. As more than one analytical choice typically varies between studies, comparisons become nontrivial. For instance, some work finds null effects between marginal or mild food insecurity and adolescent mental health but reveals a significant relationship when looking at moderate and severe levels of food insecurity (Azupogo et al., 2023; Thielman, Orr, Naraentheraraja, Harrington, & Carsley, 2024). This, however, is hard to reconcile with other contradicting evidence, where high degrees of food insecurity were not associated with adolescent mental health (Paquin et al., 2021) as these studies differ in more analytical decisions than just their hunger classifications (e.g. sample selection, covariates).

Importantly, a recent nonsystematic review suggests that on the whole, food insecurity is likely linked with worse adolescent mental health but finds that the evidence base is very heterogeneous, with measures, designs, and covariates differing widely (Dush, 2020). Taking this all together, the many plausible alternatives in analytical decisions pose challenges for direct comparison and a robust understanding of the relationship between hunger and young people's mental health.

To address these issues, here we use a principled multiverse analysis. Rather than presenting a single (or even a few) analytical model(s), a multiverse approach allows researchers to chart the entire analytical space of plausible models in an organized manner, promoting transparency and comparability (Steegen, Tuerlinckx, Gelman, & Vanpaemel, 2016). We map the effects of hunger on two types of internalizing problems: worrying and suicidality. We use the Global School‐Based Health Survey (GSHS) data, a large and diverse cross‐national survey of young people in schools. Although the GSHS does not include validated mental health measures, it does contain a single‐item measure on worrying and three items pertaining to suicidality. These data and methodological approach allow us to advance our knowledge about the impacts of hunger beyond adults and high‐income settings while transparently examining all key plausible analytical choices. Across 15,360 models, we report uncontroversially stable and significant effects of hunger on mental ill health.

## Methods

### Data and measures

We used cross‐sectional data from the GSHS, covering 410,213 adolescents across 79 countries between 2010 and 2018. We selected this time frame to avoid immediate effects of the 2008 financial crisis and the 2019 COVID‐19 pandemic. If countries were represented more than once in our period, we retained all entries. The final dataset included 96 unique country–year pairs.

Hunger and mental health were assessed using self‐reported, single‐item measures. Hunger was reported on a 5‐point scale from ‘Never’ to ‘Always’ that directly asks about the experience of hunger as linked to household food insecurity (‘During the past 30 days, how often did you go hungry because there was not enough food in your home?’). Worry was assessed on a similar 5‐point scale (‘During the past 12 months, how often were you so worried about something that you could not sleep at night?’). We treated suicidality as a broad construct and indexed it by three binary items covering ideation, planning, and attempts in the past year (‘During the past 12 months… (1) did you consider attempting suicide, (2) did you make a plan about how you would attempt suicide, (3) how many times did you attempt suicide?’). For exact survey wording on all items, see Appendix S1. We additionally included country‐level indicators of inequality and deprivation, including the Gini index, GDP, and the Multidimensional Poverty Index (MPI), matched to survey data by country and year. All data are openly and freely available online; see Appendix S2 for links and further information.

### Multiverse analysis

We report two principled multiverse analyses examining the association between hunger and worrying or suicidality. Both multiverses share the same analytical branching points, allowing us to explore all relevant, theoretically sound candidate regression models.

Our first branch considers sample selection choices by country or age, including selecting the full data or only data from countries in the same WHO‐classified region, and examining either younger or older adolescents. The second branch pertains to predictor manipulations, including no transformation or dichotomization based on different cut‐off points. We implemented min–max scaling for the continuous predictor (and outcome) measures to ensure that across all operationalizations of interest, the hunger variable lies on the same scale [0–1]. The third branch considers transformations of the outcome variable. For worrying, this means dichotomization based on different cut‐off points or the continuous measure (also min–max scaled [0–1]). For suicidality, we consider each item individually in its raw form, or take a (min–max scaled) pooled measure based on all three available items. The fourth branch considers the inclusion of different covariates, including no covariates or adjusting for individual characteristics (age and gender) or country‐level characteristics (indicators for inequality or deprivation). We standardized all covariates prior to inclusion in the model. The fifth branch allows for variation in the models' random structure, including a random intercept accounting for classroom, school, and country–year nesting in different ways. The sixth branch addresses the inclusion or exclusion of survey weights.

We have chosen our ‘base’ model specification to be a linear regression, given its robust handling of fixed and random effects, equivalent inference to logistic regressions, ability (in our chosen software) to include survey weights, and finally, straightforward interpretability (Gomila, 2021; Hellevik, 2009). The coefficients obtained from our models can be directly interpreted as a percentage point change in the probability (prevalence) of the outcome.

### Permutation control analysis

We conducted a permutation control analysis by shuffling the hunger variable across participants, reassigning each individual's reported hunger to another randomly drawn participant. We then reran the same multiverse analysis on this shuffled data. This allowed us to estimate beta‐coefficients for the same family of models as in our main analysis, meaning that these control estimates reflect the structure of the data (multilevel nesting, sampling strategies, etc.) and the analytical strategy (covariates, model specifications, etc.), but not the main predictor of interest (hunger). Thus, the control results serve as a conservative null distribution (no effect of hunger since the permuted hunger is not meaningfully associated with the corresponding individual) against which to compare our main results. We chose a permutation‐based *t*‐test over a conventional Student's *t‐*test, as the latter has an inflated false‐positive rate with very large sample sizes (i.e. more likely to obtain a statistically significant result even in the absence of a true difference between samples). Additionally, we coded whether the estimated beta‐coefficient for the main analysis was higher than the coefficient estimated in the corresponding control model (i.e. same model specification); we then ran a paired binomial test on the resulting distribution to assess statistical significance.

### Dose–response analyses

We conducted two additional multiverse analyses using multinomial logistic models to investigate the dose–response relationship between categorical hunger and worry/suicidality levels. Thus, we map the association between all possible reported levels of hunger and worry/suicidality without making assumptions about clinically meaningful thresholds or the functional form of the variable scales. Worry categories followed the original variable coding; suicidality categories reflected the sum of the three binary questions for suicidal ideation, planning, and attempts (coded as 1 for any attempts). Our multiverse analysis followed the same approach as above, mirroring the branch structure for sample and covariate selection. Note that the branches for outcome and predictor definition are not applicable here, since the multivariate regression reflects all possible levels of hunger and worry/suicidality. Due to software limitations, including survey weights and multilevel effects was not possible. We report aggregate estimates across 768 worry and 576 suicidality specifications. Our code for the multiverse, control, and dose–response analyses plus computed model summaries is on OSF: https://osf.io/ezr4x/.

## Results

We carried out two large‐scale multiverse analyses – assessing hunger's association with worry and suicidality – on data from 410,213 adolescents across 79 countries (additional descriptives are in Figures S1, S2 and Table S1). Our analyses included 15,360 unique model specifications (universes). At each branching point, we specified between 2 and 10 theoretically defensible options, many of which have already been applied in the literature (Table S2).

We found highly consistent evidence for the link between hunger and mental ill health. Experiencing hunger is associated with, on average, a 13 percentage point increase in the prevalence of experiencing worry (mean beta = 0.126, 95% CI = [0.125, 0.128], median beta = 0.12) and a 10 percentage point increase in the prevalence of experiencing suicidality (beta = 0.102, 95% CI = [0.100, 0.104], median beta = 0.07). To contextualize this, in our sample, 58% report worrying when not hungry, but 71% report worrying when hungry (13 percentage point increase or relative percent increase of about 22.4%); this corresponds to an OR = 1.79 (95% CI [1.77, 1.82]). When looking at suicidal ideation specifically, 14% report suicidal ideation when not hungry, and 19% report it when hungry (5 percentage point increase or relative percent increase of 35.7%), corresponding to an OR = 1.48 (95% CI [1.46, 1.51]).

**Figure 1:**
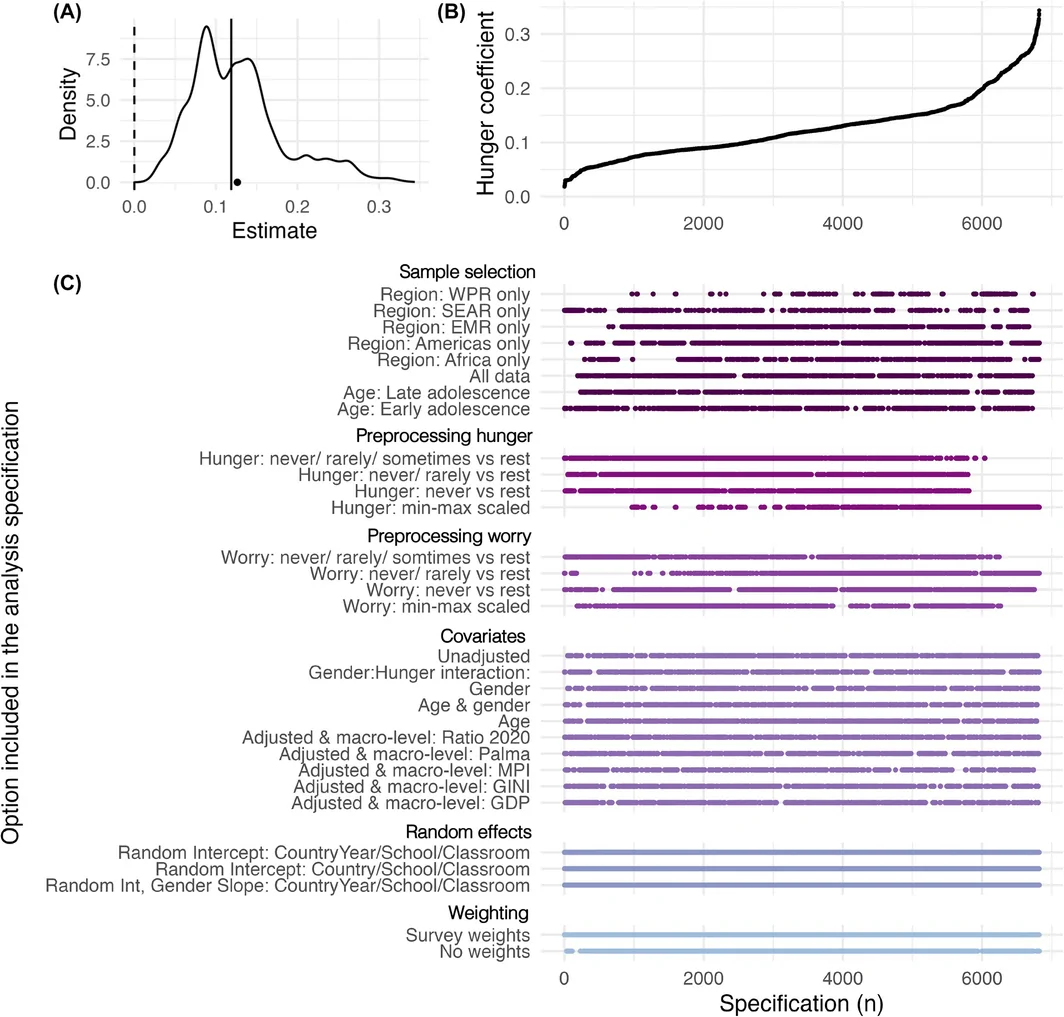
(A) Distribution of estimates of the beta‐coefficient for hunger from all model specifications predicting worry. Black line reflects the median; black circle – the mean. (B) Specification curve. Estimates of the beta‐coefficient for hunger are arranged in an ascending order by size. The x‐axis tracks universe number. (C) Model specification. The properties of the model associated with each estimate depicted in B. Colored dot reflects inclusion in model

**Figure 2:**
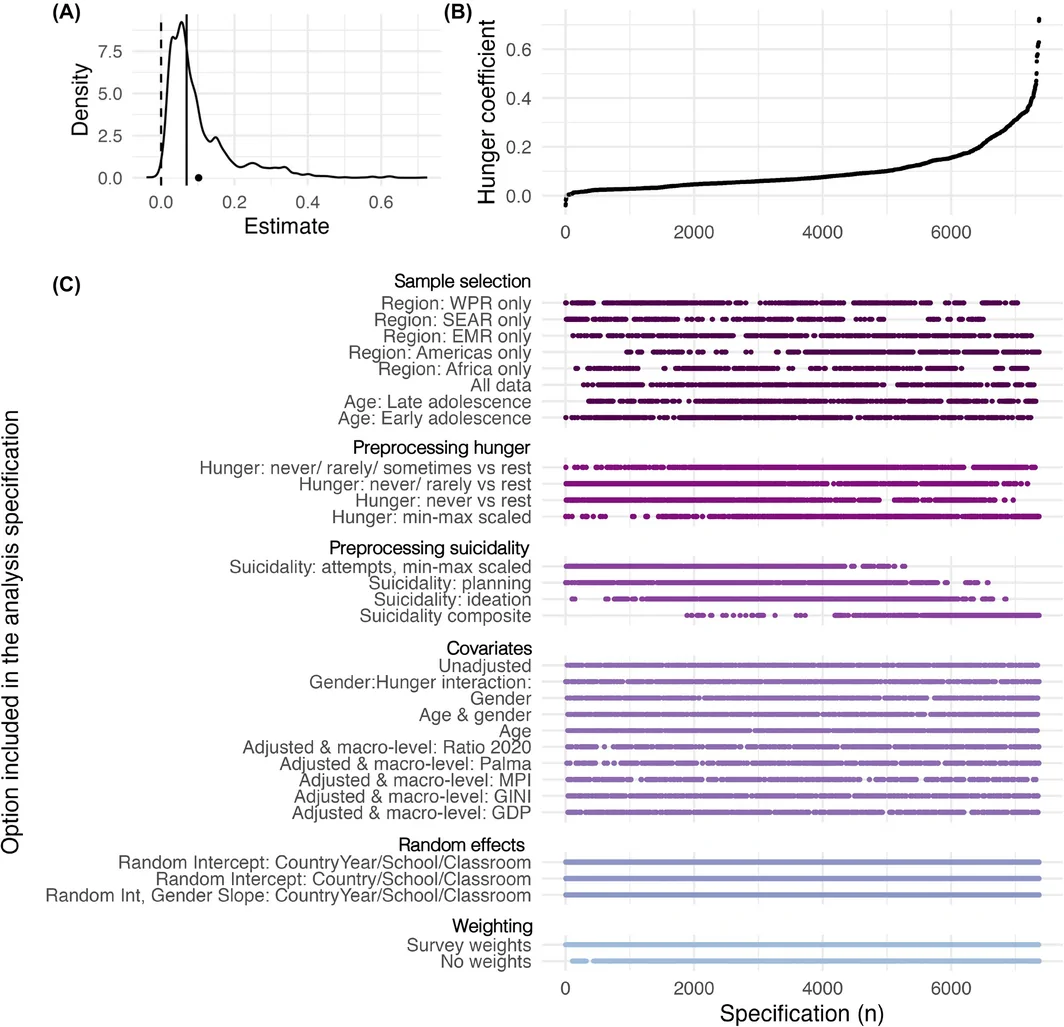
(A) Distribution of estimates of the beta‐coefficient for hunger from all model specifications predicting suicidality. Black line reflects the median; black circle – the mean. (B) Specification curve. Estimates of the beta‐coefficient for hunger are arranged in an ascending order by size. The x‐axis tracks universe number. (C) Model specification. The properties of the model associated with each estimate depicted in B. Colored dot reflects inclusion in model

**Figure 3:**
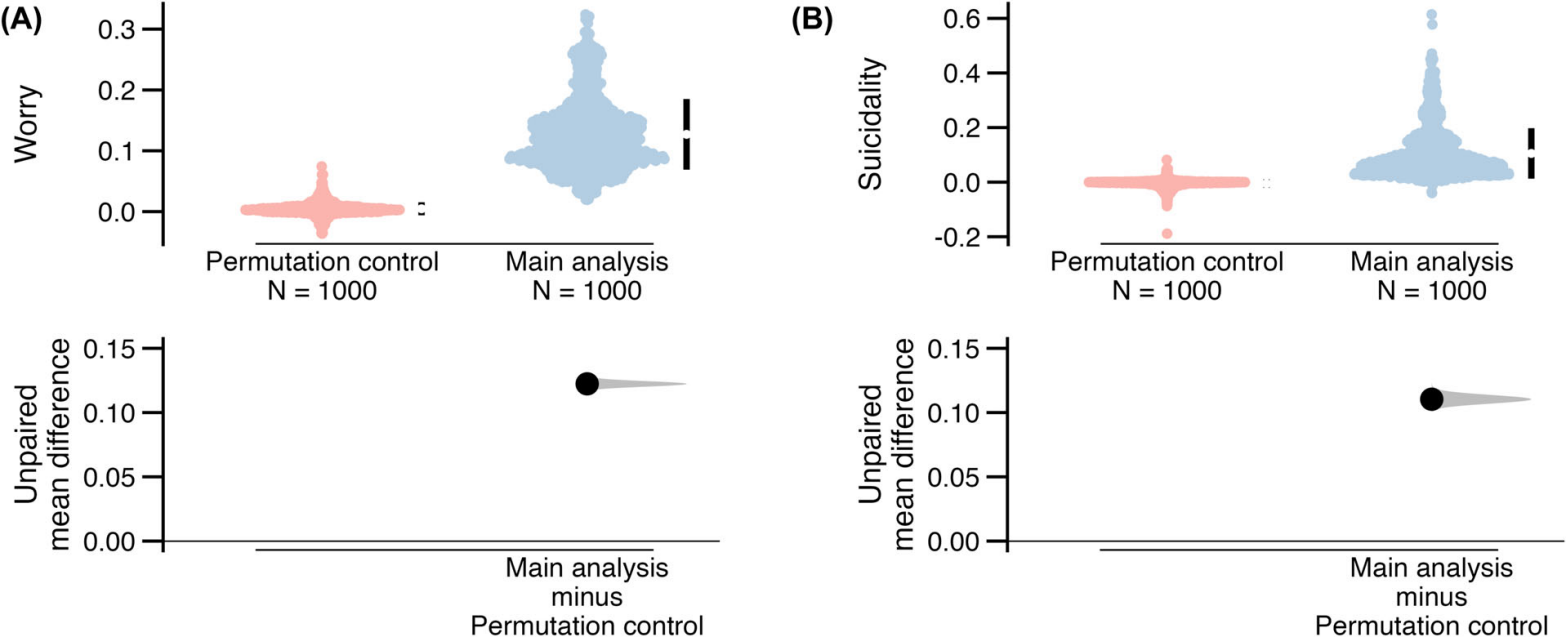
(A) Estimation plot of beta‐coefficient estimates for hunger from the permutation control analysis (red) and the main analysis (blue) on worry. Each dot is a single estimate. Black lines to the right of distributions denote standard deviation; white breaks – mean. The bottom blot depicts the results from a permutation *t*‐test and visualizes the unpaired mean difference between the estimates from the main analysis and the control analysis; the gray bell curve – the distribution of differences. The results are based on a random sample of 2000 models for computational tractability and visualization purposes. (B) As in (A) but for suicidality

This effect is strikingly robust to a variety of analytical choices. Hardly any models found no association. In the case of worry (Figure 1), we obtained a positive estimate for the effect of hunger across all specifications; in the case of suicidality (Figure 2), we obtained a positive estimate in >99% of specifications. The likelihood of obtaining these results by chance is vanishingly small, less than one in several trillion (*p* < 2.2e‐16). We further assessed our results in a frequentist statistical framework via a permutation control analysis (Figure 3). Across worry and suicidality, we obtained reliably higher beta‐coefficients at a high degree of statistical significance (worry: permutation *t*‐test *p* < 2.2e‐16, permutation difference of means = 0.12, SE = 0.001, paired binomial test *p* < 2.2e‐16, 99 + % cases main beta > control beta; suicidality: permutation *t*‐test *p* < 2.2e‐16, permutation difference of means = 0.11, SE = 0.001, paired binomial test *p* < 2.2e‐16, 97+% cases main beta > control beta). This control ensures that our results cannot be explained as a by‐product of confounds inherent in the data structure or the analytical approach.

### Variability across branches

Exploring branch‐wise variations in the estimated effect can shed light on the factors that impact the strength of the relationship between hunger and mental ill health. In Figures S3–S14, we examine the distribution of estimates across the full space of explored analytical choices. Here, we highlight several findings. Across the board, we observe a positive association between hunger and mental ill health. The effect size is largely consistent across decisions regarding sample selection, covariate selection, random effect specification, and weighting of observations, with some small exceptions. First, estimates for worry are, on average, 15%–30% higher for the Western Pacific and America regions (Western Pacific: permutation difference of means = 0.05, SE = 0.004, *p* < .2.2e‐16 and Americas: permutation difference of means = 0.02, SE = 0.002, *p* < .2.2e‐16; calculated relative to models including all data) and lower for the South‐East Asia region (permutation difference of means = −0.02, SE = 0.002, *p* < .2.2e‐16). Second, controlling for the GDP or GINI coefficient of the country decreases the effect of hunger on worrying by approximately 1 percentage point (GDP: permutation difference of means = 0.007, SE = 0.003, *p* < .05, GINI: permutation difference of means = 0.007, SE = 0.003, *p* < .05; both calculated relative to unadjusted models), suggesting that a small part of the relationship may be explained by wealth differences between and within countries (although one may argue that the wealth differences may translate to differential levels of deprivation, including hunger). Third, including survey weights boosts the strength of the effect by between half and a full percentage point (worry: permutation difference of means = 0.005, SE = 0.001, *p* < 2.2e‐16, suicidality: permutation difference of means = 0.010, SE = 0.002, *p* < 2.2e‐16). These results underscore the importance of following the correct survey weights protocol: accounting for imbalances in sampling is crucial for obtaining accurate estimates.

By far, the largest variability in the strength of the effect of hunger on mental ill health owed to choices pertaining to operationalization. This is perhaps unsurprising. If we change our standards for what counts as going hungry, we capture a different subset of the population with likely distinct corresponding mental health experiences. For suicidality, we found that the effect of hunger was stronger when we applied a conservative operationalization of hunger: counting hunger only if the adolescent reported feeling hungry most of the time or always. This strict definition of hunger increased the effect by 5 percentage points relative to applying our most liberal definition (counting hunger if the adolescent reported experiencing hunger at any point, permutation difference of means = 0.05, SE = 0.002, *p* < 2.2e‐16). These results suggest that the relationship between hunger and mental ill health may follow a dose–response pattern.

Similarly, changing how we define worry and suicidality can impact the strength of the relationship. Importantly, here, we find the opposite pattern. For both worry and suicidality, our most conservative definitions produced reliably lower estimates for the association with hunger relative to our most liberal definitions. This is, again, perhaps unsurprising: the conservative definition ignores potential meaningful mental health consequences. Counting worry only if the adolescent reported worrying most of the time or always decreased the effect of hunger by 5 percentage points relative to counting worry if the participant reported worrying at any point (permutation difference of means = 0.05, SE = 0.002, *p* < 2.2e‐16). Counting suicidality only if the adolescent attempted taking their life and scaling the number of attempts decreased the effect of hunger by 4 percentage points relative to counting suicidality if the participant reported suicidal ideation (permutation difference of means = 0.04, SE = 0.001, *p* < 2.2e‐16).

### Dose–response effects

**Figure 4:**
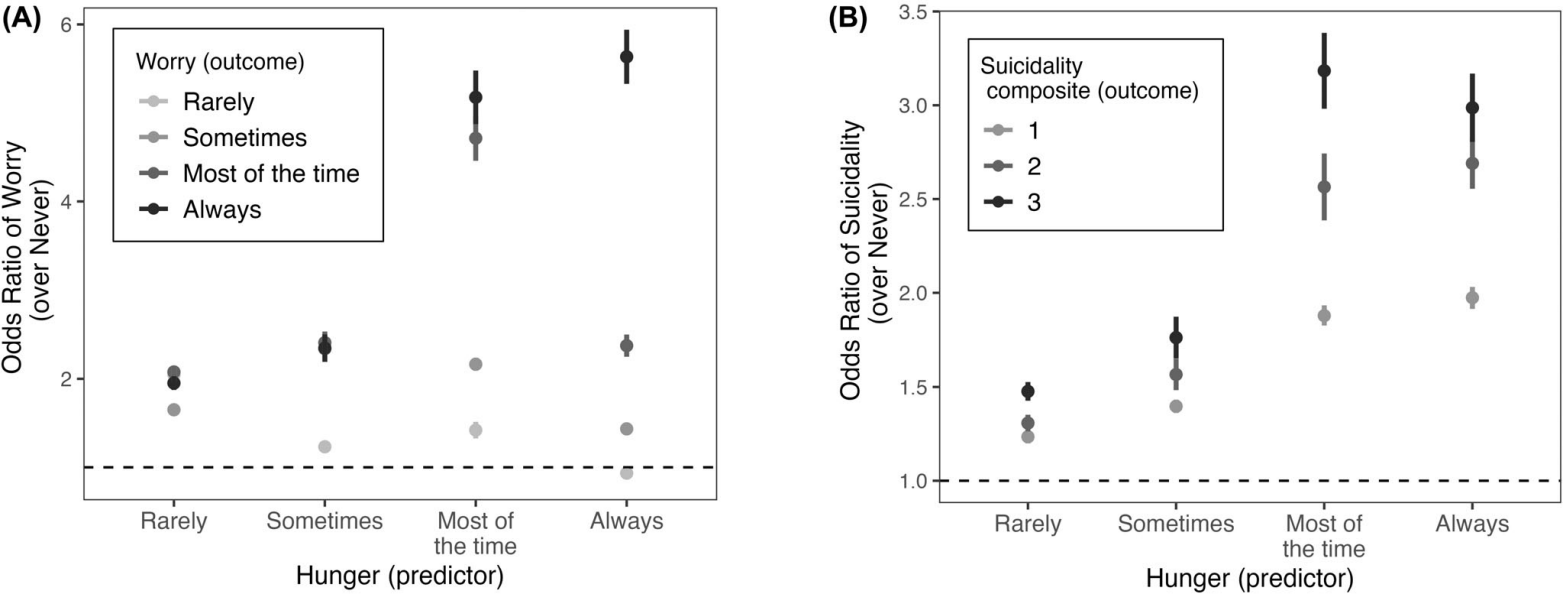
(A) Summary statistics of odds ratios from dose–response multiverse analysis on worry. The y‐axis tracks the odds ratio of reporting worry over never reporting worry. Reporting frequency is reflected by the shade of gray (lighter gray – rarely and darker gray – always). Each dot denotes the average odds ratio; the bars span the 95% confidence interval. The x‐axis tracks the hunger level (reference level = reporting never being hungry). (B) As in A, but from dose–response multiverse on suicidality. Shades of gray reflect the composite variable for suicidality aggregated from the 3 GSHS questions pertaining to suicidal ideation, planning and attempts (darker = higher; a score of 3 denotes experiencing suicidal ideation, planning, and attempt)

To investigate the dose–response relationship, we used a multinomial logistic regression, charting the relationship between each possible reported level of hunger and each possible reported level of worry/suicidality. We again followed a principled multiverse approach. The results are strikingly consistent across model specifications (Figures S15–S18, average results in Figure 4).

We find that low levels of hunger (reporting going hungry rarely or sometimes) increase the odds of experiencing any amount of worry and suicidality to a similar extent. That is, experiencing hunger rarely (as opposed to never) doubles the odds of reporting worrying (rarely, sometimes, most of the time or always) over never worrying (odds ratio = 2.05, 95% CI = [2.03, 2.07]). Similarly, experiencing hunger rarely (as opposed to never) increases the odds of reporting any suicidal ideation, planning or attempts over reporting none by 31% (odds ratio = 1.31, 95% CI = [1.30, 1.32]). By contrast, experiencing more extreme levels of hunger (reporting going hungry most of the time or always) has dissociable effects on the severity of mental ill health. In the case of worry, reporting hunger all the time increases the odds of worrying always more than fivefold (odds ratio = 5.6, 95% CI = [5.33, 5.94]) over never worrying; however, it does not make it more likely to rarely worry over never worrying (odds ratio = 0.93, 95% CI = [0.88, 0.99]). Similarly, reporting hunger all the time triples the odds of planning and attempting suicide (odds ratio = 3.18, 95% CI = [2.99, 3.39]) but doubles the odds of experiencing suicidal ideation (odds ratio = 1.97, 95% CI = [1.95, 2.00]). What this suggests is that more extreme levels of hunger are associated with more severe mental ill health, that is, a dose–response effect.

In line with this interpretation, across both worry and suicidality, we find a monotonically increasing relationship between the degree of hunger (*x*‐axis on Figure 4) and odds of reporting mental ill health (*y*‐axis on Figure 4). Strikingly, this association is stronger – i.e., the increase is steeper – for more extreme levels of mental ill health (darker shades of gray on Figure 4). This again points to a dose–response effect. Across worry and suicidality, there are indications for a saturation point of the effect: the odds ratios do not increase much beyond reporting experiencing hunger most of the time. Taken together, these results point to a consistent dose–response relationship between hunger and mental ill health in adolescents, robust to researcher degrees of freedom.

## Discussion

The relationship between hunger and adolescent mental ill health is uncontroversial: it is robust across 15,360 different model specifications, including accounting for different macro‐level measures of inequality and deprivation. It is also universally present across all geographical samples and surveyed age groups. We find that, on average, experiencing hunger is associated with a 13 percentage point increase in the prevalence of reporting worrying and a 10 percentage point increase in the prevalence of suicidality.

Using a multiverse analysis allows us to explore the full span of the magnitude of the relationship between hunger and mental health. Our specification curves for both worrying and suicidality capture effect sizes ranging from small to moderate, conceptually replicating estimates derived from various single study investigations. Importantly, we can understand why previous estimates differ. We find that the most impactful analytical choices, in terms of variability in effect size, pertain to operationalizing hunger and mental health. Our multiverse includes only operationalizations that are statistically sound, theoretically sensible, or previously motivated in the literature. Thus, we compare between plausible researcher degrees of freedom, including moral degrees of freedom, for example, different positions regarding what constitutes a notable degree of ‘risk’ (Zaneva, 2024). Taken together, this highlights the importance of transparently reporting analyses and their motivation.

There are limitations to our work. Worrying and hunger are single‐item measures, and suicidality comprises three items, all of limited validity. Together, these items broadly correspond to internalizing mental health problems, and it remains important to rigorously survey a broader range of mental health issues. Global health survey efforts should include measures with established validity and clinical relevance (see Box 1). This can be done while balancing pragmatic needs such as for brevity (e.g. with instruments like the Generalized Anxiety Disorder‐2 scale). As is common in surveys, our data comes from a single survey source, and so may be affected by same‐source bias. Moreover, we lack other relevant measures, including an understanding of desired nutrition, family socioeconomic status, and caregiver mental health, which could better contextualize the effects we observe. We attempted to address this by considering macro‐level indicators; though they operate at different levels, these measures would still capture some of the relevant variance. Further, the data analyzed here is cross‐sectional and cannot support causal inferences.

Understanding causal direction and mechanisms is crucial for effective interventions. Possible mechanisms have been proposed in the literature. One possible mechanism implicates the role of nutritional deficiencies as part of going hungry: diets lacking essential vitamins and minerals can lead to anxiety and depression (Kaplan, Crawford, Field, & Simpson, 2007). Another involves inflammation, whereby healthy foods mitigate inflammation, but lack of such foods can heighten risk (Clesse, Yaqoob, Jayakumar, Bhui, & Carvalho, 2022). Finally, hunger and food insecurity can create profound uncertainty and stress about nutrition, health, and capacity; both uncertainty and stress (including allostatic load) have been evidenced as risk factors for internalizing problems (McEwen, 2004). Crucially, for children and adolescents reverse causality accounts – where mental health problems lead to planning or motivational deficits which in turn can explain the experience of hunger – are less plausible. This is because the socioeconomic status and food security of younger cohorts tend to be determined by their caregivers (Zaneva, Dumbalska, Reeves, & Bowes, 2024). Direct causal proof, however, is difficult to obtain because it is unconscionable to randomize children to experience hunger or deny them available support. Future research must instead rely on ethically designed interventions to solidify this evidence base. However, such evidence is critically lacking: a recent systematic review focusing on sub‐Saharan Africa found no interventions that investigated the effect of reducing food insecurity on the mental health of children and adolescents (Kasujja, Lund, & Salisbury, 2025). Such interventions are much needed and can take inspiration from the design of cash transfer trials that assess mental health (Zaneva, Guzman‐Holst, Reeves, & Bowes, 2022) – for instance, randomized controlled trials where food insecure young people are provided with direct, unconditional food support, their mental health is assessed at both baseline and follow‐up, and compared with control arm adolescents who are on a short waitlist.

As we took an exploratory approach, we did not preregister a minimum effect size of interest. It is nevertheless important to contextualize the effects we report here. A recent study of adults from the UK and France found that food insecure status was linked to worse mental health with odds ratios of approximately 1.17 (combined measure of depression and anxiety) to 1.23 (for anxiety) (Bateson et al., 2025). A meta‐analysis of adults, primarily in high‐income countries, showed a positive relationship between food insecurity and risk for depression (OR = 1.40; 95% [CI: 1.30, 1.58]) and stress (OR = 1.34; 95% CI: [1.24, 1.44]) but not anxiety. The effects we report here on the relationship of subjective hunger due to lacking food at home and worrying and suicidality, based on a global sample of children and adolescents, are slightly higher. We find that experiencing hunger is linked with, on average, a 13 percentage point increase in the prevalence of worrying, and a 10 percentage point increase in the prevalence of suicidality, corresponding to a sample‐wide OR = 1.79 (95% CI [1.77, 1.82]) for worrying and OR = 1.48 (95% CI [1.46, 1.51]) for suicidal ideation. This points to the great importance of better surveying and understanding the social protection needs of adolescents worldwide. Given the nationally representative data surveyed here, our effects are not only important given their nontrivial magnitude, but also because they likely operate at a population level (Rose, 1985). This substantiates the need for effective population‐level interventions that address food security, as these would yield greater health benefit than targeting small segments of the population.

Further, to the best of our knowledge, we offer what is the most consistent evidence for a relationship between mental health and any predictor. Previous large‐scale multiverse analyses into mental health and well‐being showed mixed findings for the impacts of alcohol (Halladay et al., 2024) personal traits like self‐esteem (Peetz, Lansu, van den Berg, & Cillessen, 2024), inflammation markers (Rengasamy, Moriarity, Kraynak, Tervo‐Clemmens, & Price, 2023), digital technology use (Orben & Przybylski, 2019) and traumatic events (McGuire & Jackson, 2024). In other words, not only are our findings critically important in the context of the developing literature on hunger and mental health, but they are also fundamental in understanding what the most stable, uncontroversial predictors for mental health are. The experience of hunger, especially when it is frequent, is one of the most uncontestably clear predictors of mental ill health.

In terms of implications, our results are in correspondence to the Sustainable Development Goals (1, 2, 3) on the eradication of food insecurity and poverty. Importantly, as mentioned previously, since the pronounced and robust effect of hunger likely operates on a population level (Pearce, 2000) even small improvements in population‐level effects can produce greater total benefit than large changes at the individual level (Carey, Ridler, Ford, & Stringaris, 2023; Rose, 1985). Population‐level interventions should be promoted, such as universal free school meals (Box 1). Universal screening for food security should be supported in clinical settings, in line with recommendations by clinical organizations such as the American Academy of Pediatrics (Kumar et al., 2025). Further, integrating mental health assessments into food assistance programs is essential for safeguarding particularly vulnerable adolescents and families and those facing severe or chronic food insecurity, given the pronounced dose–response effect. Similarly, mental health assessments should form part of food assistance and crisis response planning during public health emergencies, given emerging evidence from the COVID‐19 pandemic for an exacerbation of the impacts of pandemic‐caused food insecurity on mental health (Fang, Thomsen, & Nayga, 2021).

Current estimates suggest that almost a billion people will face severe food insecurity risk by 2030 (Fu, Shetty, Carmichael, & Andree, 2025). These economic burdens are already staggering: a recent report indicated that market failures and inefficiencies in the global food system are linked with over $10 trillion in costs (Fu et al., 2025). Given that estimates of the costs of hunger do not consistently factor in the mental health outcomes of young people, who are the next generation to enter the workforce, we are likely currently vastly underestimating the economic and social costs of hunger. Crucially, there is enough food for everyone living on our planet today (UN Environment Programme, 2020). The remarkable societal and financial losses we are witnessing expose the clear need for systemic changes to improve the efficiency and equity of food distribution.

Box 1 **Summary of research and policy implications**

**Table:** 

Research implications	There is a need for higher measurement standards in global health survey and monitoring efforts, including: Use of validated mental health measures Inclusion of wider range of measures to capture varied aspects of mental health (e.g. internalizing and externalizing problems) Use of validated measures for food insecurity Inclusion of wider range of measures to capture varied aspects of food insecurity (e.g. experience of hunger, dietary diversity and preferences, nutritional status, caloric intake etc.) Use of culturally and contextually sensitive and adapted measures There is a need for stronger empirical designs to understand the mental health of adolescents globally, including Appropriate data to better understand and directly assess potential causal mechanisms Longitudinal data, with ability to link individual observations, to track changes in food insecurity and health over time, as well as understand potential long‐term impacts of chronic versus more transient food insecurity Ethically designed interventions, where the effect of reducing hunger and food insecurity on mental health is assessed
Policy implications	Addressing food insecurity, and the equitable and efficient distribution of food worldwide, are relevant considerations for comprehensive adolescent mental health protection and suicide prevention Given the population‐level effect of hunger, population‐level interventions should be considered such as for instance, universal free school (or community) meal programs Food security screening in clinical settings should also be supported Assessing food security should form part of holistic crisis response planning (e.g. during public health emergencies, environmental disasters, or economic shocks) Mental health screening can be adopted in food assistance programs, particularly for vulnerable adolescents and families Given the evidence for a dose–response relationship, particular attention should be paid to children and adolescents at risk of experiencing high levels of food insecurity

## Ethical considerations

Ethical approval was not applicable as this study uses already existing, anonymized secondary data.

**Key points**

**What's known?**

- While much research has focused on the physiological and objective dimensions of food insecurity, the subjective experience of hunger and its impact on mental health remains remarkably underexplored in behavioral science. This is particularly the case for adolescents, and those living in low‐ and middle‐income contexts.

**What's new?**

- Previous work is mixed, especially for younger populations, where effects vary both in magnitudes and in statistical significance. Through a principled multiverse analysis of large‐scale global data from 79 countries and 410,213 adolescents, our work demonstrates a remarkably stable negative association between hunger and mental health.
- Our multiverse analysis includes 15,360 model specifications, thus allowing us to systematically account for various plausible analytical choices and offer what is, to our knowledge, the most robust and convincing evidence for any predictor of mental health.

**What's relevant?**

- Taking all evidence into account, our work supports the notion that addressing food insecurity, and the equitable and efficient distribution of food worldwide, is as an important and relevant consideration for adolescent mental health protection and suicide prevention.

## Supporting information


**Appendix S1.** GSHS survey questions.
**Appendix S2.** Data access.
**Table S1.** Descriptive statistics per country.
**Table S2.** Multiverse branches.
**Figure S1.** Distributions of reported worry, suicidality, and hunger by gender.
**Figure S2.** Histograms of worry, age, and hunger levels.
**Figure S3.** Results of worry multiverse by sample selection.
**Figure S4.** Results of worry multiverse by predictor preprocessing.
**Figure S5.** Results of worry multiverse by outcome preprocessing.
**Figure S6.** Results of worry multiverse by covariate selection.
**Figure S7.** Results of worry multiverse by random structure.
**Figure S8.** Results of worry multiverse by sampling weights.
**Figure S9.** Results of suicidality multiverse by sample selection.
**Figure S10.** Results of suicidality multiverse by predictor preprocessing.
**Figure S11.** Results of suicidality multiverse by outcome preprocessing.
**Figure S12.** Results of suicidality multiverse by covariate selection.
**Figure S13.** Results of suicidality multiverse by random structure.
**Figure S14.** Results of suicidality multiverse by sampling weights.
**Figure S15.** Results of worry dose–response multiverse by sample selection.
**Figure S16.** Results of worry dose–response multiverse by covariate selection.
**Figure S17.** Results of suicidality dose–response multiverse by sample selection.
**Figure S18.** Results of suicidality dose–response multiverse by covariate selection.

## Data Availability

This project uses openly available data. The GSHS survey is available in the NCD Microdata Repository at: https://extranet.who.int/ncdsmicrodata/index.php/catalog/GSHS/ Our country‐level indicators are available from the Standardized World Income Inequality Database at: https://fsolt.org/swiid/ (Gini index), the World Income Inequality Database at: https://www.wider.unu.edu/database/world‐income‐inequality‐database‐wiid (Palma ratio, the 20/20 ratio, GDP per capita), and the Oxford Poverty and Human Development Initiative at: https://ophi.org.uk/global‐mpi (MPI).
