# Supplementary material for: Hunger as an uncontroversial predictor of poor adolescent mental health: evidence from a multiverse analysis of 410,213 adolescents across 79 countries
Source: J Child Psychol Psychiatry. 2026 Jun 2;67(10):1741–51. doi: 10.1111/jcpp.70176 (PMC13535740; doi:10.1111/jcpp.70176)
Supplement: Supplementary file 1 [file JCPP-67-1741-s001.docx]

**Hunger as an uncontroversial predictor of poor adolescent mental health: evidence from a multiverse analysis of 410,213 adolescents across 79 countries**

**Supporting Information**

Contents

1. Appendix S1: GSHS survey questions.
2. Appendix S2: Data access.
3. Table S1: Descriptive statistics per country.
4. Table S2: Multiverse branches.
5. Figure S1: Distributions of reported worry, suicidality and hunger by gender.
6. Figure S2: Histograms of worry, age and hunger levels.
7. Figure S3: Results of worry multiverse by sample selection.
8. Figure S4: Results of worry multiverse by predictor preprocessing.
9. Figure S5: Results of worry multiverse by outcome preprocessing.
10. Figure S6: Results of worry multiverse by covariate selection.
11. Figure S7: Results of worry multiverse by random structure.
12. Figure S8: Results of worry multiverse by sampling weights.
13. Figure S9: Results of suicidality multiverse by sample selection.
14. Figure S10: Results of suicidality multiverse by predictor preprocessing.
15. Figure S11: Results of suicidality multiverse by outcome preprocessing.
16. Figure S12: Results of suicidality multiverse by covariate selection.
17. Figure S13: Results of suicidality multiverse by random structure.
18. Figure S14: Results of suicidality multiverse by sampling weights.
19. Figure S15: Results of worry dose-response multiverse by sample selection.
20. Figure S16: Results of worry dose-response multiverse by covariate selection.
21. Figure S17: Results of suicidality dose-response multiverse by sample selection.
22. Figure S18: Results of suicidality dose-response multiverse by covariate selection.

**Appendix S1: GSHS survey questions**

***GSHS Measures:*** We report the exact wording from GSHS.

| **Construct** | **GSHS Module** | **Question** | **Response options** |
| --- | --- | --- | --- |
| Age | Demographics Module | 1. How old are you? | A. 11 years old or younger  B. 12 years old  C. 13 years old  D. 14 years old  E. 15 years old  F. 16 years old  G. 17 years old  H. 18 years old or older |
| Sex | Demographics Module | 2. What is your sex? | A. Male  B. Female |
| Hunger | Dietary Behaviours Module | 3. During the past 30 days, how often did you go hungry because there was not enough food in your home? | A. Never  B. Rarely  C. Sometimes  D. Most of the time  E. Always |
| Worry | Mental Health | 3. During the past 12 months, how often were you  so worried about something that you could not  sleep at night? | A. Never  B. Rarely  C. Sometimes  D. Most of the time  E. Always |
| Suicidality (consider) | Mental Health | 4. During the past 12 months, did you seriously  consider attempting suicide? | A. Yes  B. No |
| Suicidality (plan) | Mental Health | 5. During the past 12 months, did you make a plan about how you would attempt suicide? | A. Yes  B. No |
| Suicidality (attempt) | Mental Health | 6. During the past 12 months, how many times did you attempt suicide? | A. 0 times  B. 1 time  C. 2 or 3 times  D. 4 or 5 times  E. 6 or more times |

**Appendix S2: Data access**

1. **GSHS data**

The Global School-based Health Survey (GSHS) is a collaborative international survey that can be accessed free of charge.

For information about the GSHS: <https://www.who.int/teams/noncommunicable-diseases/surveillance/systems-tools/global-school-based-student-health-survey>

For download navigate to the NCD Microdata Repository <https://extranet.who.int/ncdsmicrodata/index.php/home>

Where GSHS is directly found: <https://extranet.who.int/ncdsmicrodata/index.php/catalog/GSHS/?page=1&ps=15&repo=GSHS>

2. **Macro-level indicators**

We included country-level measures for different facets inequality or deprivation. For our macro-level inequality measures, we used the Standardized World Income Inequality Database to download the Gini index: <https://fsolt.org/swiid/>

We downloaded the Palma ratio and the 20/20 ratio, GDP per capita from the World Income Inequality Database: <https://www.wider.unu.edu/database/world-income-inequality-database-wiid>

We downloaded the Global Multidimensional Poverty Index (MPI), a composite measure of deprivation assessing indicators for health, education, and living standards, from the Oxford Poverty and Human Development Initiative: <https://ophi.org.uk/global-mpi>

At present, all data used here is freely available and directly accessible from the mentioned sources.

**Table S1: Descriptive statistics per country**

For each country-year in our specified period we provide the mean age, the proportion of girls, of reported hunger, of worrying, and of suicidality. Countries can opt out of certain questions.

| **Country** | **Year** | | **Age** | | **Girls** | | **Hunger** | | **Worry** | **Suicidality** |
| --- | --- | --- | --- | --- | --- | --- | --- | --- | --- | --- |
| Afghanistan | 2014 | 15.09 | | 0.58 | | 0.49 | | 0.63 | | 0.19 |
| Algeria | 2011 | 14.12 | | 0.52 | | 0.46 | | NA | | NA |
| Anguilla | 2016 | 14.71 | | 0.52 | | 0.44 | | 0.61 | | 0.23 |
| Argentina | 2012 | 14.51 | | 0.52 | | 0.37 | | 0.64 | | 0.18 |
| Argentina | 2018 | 14.91 | | 0.52 | | 0.32 | | 0.71 | | 0.22 |
| Bahamas | 2013 | 13.41 | | 0.54 | | 0.48 | | 0.59 | | 0.18 |
| Bahrain | 2016 | 14.13 | | 0.48 | | 0.55 | | 0.67 | | 0.14 |
| Bangladesh | 2014 | 14.2 | | 0.6 | | 0.64 | | 0.6 | | 0.04 |
| Barbados | 2011 | 14.18 | | 0.55 | | 0.42 | | 0.54 | | NA |
| Belize | 2011 | 13.83 | | 0.53 | | 0.37 | | 0.6 | | 0.14 |
| Benin | 2016 | 16.38 | | 0.46 | | 0.55 | | 0.72 | | 0.15 |
| Bhutan | 2016 | 15.64 | | 0.55 | | NA | | 0.72 | | 0.12 |
| Bolivia | 2012 | 14.36 | | 0.49 | | 0.63 | | 0.71 | | 0.19 |
| Bolivia | 2018 | 15.45 | | 0.48 | | 0.56 | | 0.72 | | 0.22 |
| Brunei Darussalam | 2014 | 14.64 | | 0.53 | | 0.61 | | 0.71 | | 0.1 |
| Cambodia | 2013 | 15.65 | | 0.53 | | 0.54 | | 0.73 | | 0.07 |
| Chile | 2013 | 14.65 | | 0.5 | | 0.28 | | NA | | NA |
| Cook Islands | 2011 | 14.44 | | 0.52 | | 0.66 | | 0.61 | | NA |
| Cook Islands | 2015 | 15.37 | | 0.51 | | 0.69 | | 0.65 | | 0.15 |
| Curacao | 2015 | 15.25 | | 0.55 | | 0.25 | | 0.59 | | 0.11 |
| Dominican Republic | 2016 | 14.91 | | 0.56 | | 0.34 | | 0.63 | | 0.18 |
| Egypt | 2011 | 13.52 | | 0.53 | | 0.46 | | NA | | NA |
| El Salvador | 2013 | 14.33 | | 0.46 | | 0.35 | | 0.6 | | 0.14 |
| Eswatini | 2013 | 16.04 | | 0.52 | | 0.54 | | 0.54 | | 0.17 |
| Fiji | 2010 | 14.32 | | 0.57 | | 0.66 | | 0.66 | | 0.18 |
| Fiji | 2016 | 15.79 | | 0.51 | | 0.6 | | 0.59 | | 0.13 |
| French Polynesia | 2015 | 15.05 | | 0.54 | | 0.64 | | 0.64 | | 0.15 |
| Ghana | 2012 | 15.99 | | 0.46 | | 0.65 | | 0.67 | | 0.19 |
| Guatemala | 2015 | 14.36 | | 0.51 | | 0.38 | | 0.63 | | 0.21 |
| Honduras | 2012 | 13.91 | | 0.52 | | 0.36 | | 0.63 | | 0.19 |
| Indonesia | 2015 | 14.03 | | 0.54 | | 0.55 | | 0.53 | | 0.06 |
| Iraq | 2012 | 14.32 | | 0.44 | | 0.35 | | 0.63 | | 0.17 |
| Jamaica | 2010 | 14.59 | | 0.51 | | 0.56 | | 0.6 | | 0.22 |
| Jamaica | 2017 | 15.02 | | 0.54 | | 0.49 | | 0.58 | | 0.26 |
| Kiribati | 2011 | 14.32 | | 0.56 | | 0.67 | | 0.56 | | 0.35 |
| Kuwait | 2011 | 14.35 | | 0.5 | | 0.38 | | 0.66 | | 0.2 |
| Kuwait | 2015 | 15.12 | | 0.53 | | 0.49 | | 0.73 | | 0.17 |
| Lao People's Democratic Republic | 2015 | 15.62 | | 0.54 | | 0.5 | | 0.66 | | 0.03 |
| Lebanon | 2011 | 13.91 | | 0.53 | | 0.35 | | 0.63 | | 0.15 |
| Lebanon | 2017 | 14.87 | | 0.59 | | 0.29 | | 0.68 | | 0.14 |
| Liberia | 2017 | 16.64 | | 0.48 | | 0.69 | | 0.76 | | 0.27 |
| Malaysia | 2012 | 14.91 | | 0.5 | | 0.6 | | 0.61 | | 0.07 |
| Maldives | 2014 | 15.51 | | 0.58 | | 0.38 | | 0.68 | | 0.14 |
| Mauritania | 2010 | 14.76 | | 0.53 | | 0.59 | | 0.55 | | 0.18 |
| Mauritius | 2011 | 13.9 | | 0.54 | | 0.26 | | NA | | NA |
| Mauritius | 2017 | 14.87 | | 0.53 | | 0.42 | | 0.61 | | 0.16 |
| Mongolia | 2010 | 14.67 | | 0.57 | | 0.34 | | 0.57 | | 0.2 |
| Mongolia | 2013 | 14.53 | | 0.53 | | 0.37 | | 0.58 | | 0.23 |
| Morocco | 2010 | 13.98 | | 0.48 | | 0.32 | | 0.54 | | 0.17 |
| Morocco | 2016 | 14.83 | | 0.47 | | 0.34 | | 0.6 | | 0.16 |
| Mozambique | 2015 | 15.89 | | 0.47 | | 0.47 | | 0.65 | | 0.19 |
| Myanmar | 2016 | 14.25 | | 0.54 | | 0.32 | | 0.48 | | 0.09 |
| Namibia | 2013 | 15.77 | | 0.53 | | 0.54 | | 0.64 | | 0.2 |
| Nauru | 2011 | 14.32 | | 0.58 | | 0.72 | | 0.74 | | 0.29 |
| Nepal | 2015 | 14.5 | | 0.53 | | 0.35 | | 0.51 | | 0.13 |
| Niue | 2010 | 14.23 | | 0.41 | | 0.6 | | NA | | 0.12 |
| Occupied Palestinian Territory | 2010 | 13.87 | | 0.54 | | 0.37 | | 0.69 | | 0.2 |
| Oman | 2010 | 14.87 | | 0.53 | | 0.42 | | NA | | NA |
| Oman | 2015 | 15.42 | | 0.52 | | 0.38 | | 0.72 | | 0.22 |
| Panama | 2018 | 15.35 | | 0.55 | | 0.42 | | 0.74 | | 0.19 |
| Paraguay | 2017 | 14.85 | | 0.52 | | 0.29 | | 0.66 | | 0.14 |
| Peru | 2010 | 14.46 | | 0.51 | | 0.52 | | 0.78 | | 0.2 |
| Phillippines | 2011 | 14.5 | | 0.57 | | 0.65 | | 0.77 | | 0.17 |
| Phillippines | 2015 | 14.62 | | 0.54 | | 0.7 | | 0.76 | | 0.1 |
| Qatar | 2011 | 13.31 | | 0.54 | | 0.31 | | 0.59 | | NA |
| Saint Kitts and Nevis | 2010 | 14.35 | | 0.56 | | 0.41 | | 0.6 | | 0.17 |
| Saint Lucia | 2018 | 14.36 | | 0.53 | | 0.51 | | 0.59 | | 0.25 |
| Saint Vincent and the Grenadines | 2018 | 15.33 | | 0.53 | | 0.54 | | 0.64 | | 0.27 |
| Samoa | 2011 | 14.04 | | 0.59 | | 0.81 | | 0.72 | | 0.33 |
| Samoa | 2017 | 15.07 | | 0.63 | | 0.73 | | 0.57 | | 0.23 |
| Seychelles | 2015 | 13.84 | | 0.53 | | 0.44 | | 0.61 | | 0.22 |
| Sierra Leone | 2017 | 15.32 | | 0.54 | | NA | | 0.65 | | 0.15 |
| Solomon Islands | 2011 | 14.59 | | 0.48 | | 0.83 | | 0.7 | | 0.3 |
| Sri Lanka | 2016 | 14.7 | | 0.56 | | 0.28 | | 0.45 | | 0.09 |
| Sudan | 2012 | 14.84 | | 0.61 | | 0.43 | | NA | | NA |
| Suriname | 2016 | 14.79 | | 0.51 | | 0.43 | | 0.53 | | 0.16 |
| Syrian Arabic Republic | 2010 | 13.65 | | 0.6 | | 0.53 | | 0.72 | | NA |
| Thailand | 2015 | 14.5 | | 0.57 | | 0.56 | | 0.68 | | 0.12 |
| Timor-Leste | 2015 | 15.39 | | 0.54 | | 0.51 | | 0.52 | | 0.1 |
| Tokelau | 2014 | 14.07 | | 0.52 | | 0.65 | | 0.61 | | 0.26 |
| Tonga | 2010 | 14.19 | | 0.54 | | 0.74 | | 0.65 | | NA |
| Tonga | 2017 | 14.21 | | 0.54 | | 0.65 | | 0.55 | | 0.12 |
| Trinidad and Tobago | 2011 | 13.61 | | 0.45 | | 0.49 | | 0.48 | | 0.17 |
| Trinidad and Tobago | 2017 | 14.18 | | 0.53 | | 0.52 | | 0.57 | | 0.22 |
| Tuvalu | 2013 | 13.97 | | 0.51 | | 0.53 | | 0.51 | | 0.08 |
| United Arab Emirates | 2010 | 14.09 | | 0.58 | | 0.3 | | 0.73 | | 0.16 |
| United Arab Emirates | 2016 | 14.99 | | 0.52 | | 0.49 | | 0.67 | | 0.15 |
| United Republic of Tanzania | 2014 | 14.26 | | 0.52 | | 0.26 | | 0.4 | | 0.14 |
| Uruguay | 2012 | 14.43 | | 0.54 | | 0.24 | | 0.6 | | 0.12 |
| Vanuatu | 2011 | 13.78 | | 0.56 | | 0.53 | | 0.53 | | 0.18 |
| Vanuatu | 2016 | 14.98 | | 0.56 | | 0.62 | | 0.54 | | 0.15 |
| Viet Nam | 2013 | 15.58 | | 0.53 | | 0.52 | | NA | | 0.16 |
| Wallis and Fortuna | 2015 | 14.6 | | 0.52 | | 0.63 | | 0.6 | | 0.23 |
| Yemen | 2014 | 14.82 | | 0.51 | | 0.57 | | 0.68 | | 0.16 |
| Zimbabwe | 2013 | 14.74 | | 0.55 | | NA | | 0.64 | | 0.39 |

**Table S2: Multiverse branches**

In the column ‘Branch’ we list what each branch in our multiverse considers. In the second column, we provide the specific branch options we consider in our multiverse analysis. In the third column, we offer some examples of different choices that exist in the literature. All examples are from work with the same dataset, the Global school-based student health survey (GSHS). Finally, we list general considerations which can guide the use or non-use of certain analytical choices. This Table is not exhaustive but intended to illustrate the wide variety of plausible analytical choices.

| **Branch** | **Options in our multiverse** | **Examples of different choices in literature** | **General considerations** |
| --- | --- | --- | --- |
| Covariates | No covariates or adjusting for individual characteristics (age and gender) or country-level characteristics (indicators for inequality and deprivation) | Individual-level factors such as age and gender are commonly considered (Kerkadi et al., 2021).  Country level covariates can include various indicators such as, among others, the Poverty Headcount Ration, Squared or non-squared Poverty Gap, monthly household per capita income (Chen et al., 2024), the Gender Inequality Index, Gross Domestic Product, Global Food Security Index (Candler et al., 2016), and some do not account for country-level factors (Pengpid & Peltzer, 2024). | Covariate selection can be motivated theoretically with a causal inference view, a view of assessing confounds or mediating variables. Statistically, considerations related to model fit (including overfitting) and explanatory power, precision and predictive ability. Practical motivations can pertain to data availability, sample size, parsimony, missingness. Researchers may consider some covariates in correspondence with existing literature. |
| Predictor | No transformation of hunger item, dichotomization based on different cut-offs | Hunger is considered in raw form, as a 5-point scale (Probst et al., 2020). Transformations are varied, dichotomizations include Never = 0 and all other responses = 1 (Wight, et al., 2014); Never and Rarely = 0, Sometimes, Most of the time and Always = 1 (Liu, et al., 2022), Never, Rarely, Sometimes = 0, Most of the time and Always = 1 (Mwanmebe, Muula, & Leo, 2013). Some trichotomize, for instance such that Never = 1, Rarely and Sometimes = 1, and Most of the Time and Always = 2 (Smith et al., 2020) | Transformation decisions can be given the assumptions of statical models, with considerations about linearity, normality, homoscedasticity, as well as considerations regarding improving model fit, reducing outlier impact, simplifying interpretation or maintaining consistent interpretation scales across other works and measures. Some fields may have specific norms or the original manual for scales may recommend certain transformations (e.g. dichotomization based on diagnostic cut-off). |
| Random structure | Random intercept for classroom, school and country year nesting in different ways, as well as considering a random slope for gender | Random structures considered in the literature include a random intercept (Shadmehr et al., 2021; Noel 2019), as well as random effects accounting for different levels (classroom, school, country) and for gender, hunger (Wilson, Dunlavy, & Berchtold, 2013). Models without random structures are also used (Beck et al., 2013; Chen et al., 2024). | The choice of random structure for a model can be guided by theoretical, statistical, practical and data-driven motivations such as regarding the data’s hierarchical nature, presence of unobserved heterogeneity, generalizability, model fit and parsimony, computational feasibility. |
| Sample selection | Selecting full data, or only data for countries in the same WHO-classified region, or only for younger or older adolescents | Sampling by countries includes decisions such as capturing low- and middle-income countries only (Liu, Huang, & Liu, 2018; Uddin et al., 2019), focusing on a particular continent, region or area (Caribbean – Pengpid & Peltzer, 2020; Latin America, da Silva et al., 2024; Polynesian countries, Denny et al., 2016), and capturing all countries with data where the variables of interests are included (Steare et al., 2023). Age can be a factor in sampling decisions, such as for instance selecting a wider age range to allow comparisons between developmental periods (younger 13-15 year olds compared to older adolescents 16-17 year olds; Campisi et al., 2020). | Sampling decisions can be made with regards to conceptual focus, representativeness, possibility for comparative analyses, homogeneity, data validity, resources constraints, sample size and power, bias reduction, confidentiality (certain data may be restricted). |
| Weights | Use or non-use of sampling weights | The GSHS provided sampling weights have been used by some researchers (Brown et al., 2009; Beal et al., 2019). Others have calculated weights on their own in different ways (Han et al., 2019; Dema et al., 2019; Van Minh et al., 2011). Some have not used weights (Ahinkorah et al., 2021; Yusuf et al., 2019). | The use or non-use of weights can be motivated theoretically regarding representativeness, exchangeability; statistically regarding bias reduction, estimation precision, generalizability; and practically regarding the type of data, sampling design, nonresponse adjustments, bias reduction. |
| Outcome | For worrying, considering it in raw or binary form  For suicidality, considering each available item on their own in raw form, or taking a pooled measure based on all three items | Binary worry transformation options include Most of the time and Always = 1, Never, Rarely, Sometimes= 0 (Wang, 2022); Never and Rarely = 0, and Sometimes, Most of the time, Always = 0 (Wang, Liu, Mati, 2020). Raw/ untransformed worry is also used (Kearns et al., 2021). For suicidality, items have been considered on their own as separate outcomes; sometimes all three items are considered on their own (Steare et al., 2023; Baiden et al., 2019), sometimes only one item is examined (e.g. considered suicide, Johnson et al., 2019). Suicide attempts have been dichotomized, such that no attempts = 0, and 2 to 6 attempts= 1 (Tetteh et al., 2021). One pooled total suicide behavior measure is binary, assessing whether students reported any form of suicidal ideation, planning or attempts (Khan et al., 2020; Marthoenis & Arafat, 2022). | See above regarding transformation decisions. Pooling items together can be motivated regarding construct validity, construct definition and comparison with other literature. Measurement considerations relate to reliability, validity, measurement error, ease of analysis. |

**Figure S1. Distributions of reported worry, suicidality and hunger by gender**

**A.** Estimation plot of average levels of worry (coded 0 if the adolescent reported never worrying and 1 otherwise). Each dot is a country-year pair (green – girls, purple – boys). The distribution on the right depicts the mean difference between boys and girls (distribution, mean and standard deviation of the difference from a permutation-based t-test). Girls report higher levels of worrying, on average. **B.** As in A, but for suicidal ideation. Boys report higher levels of suicidality, on average. **C.** As in A, but for reported hunger (coded 0 if the adolescent reported never worrying and 1 otherwise). Reported hunger did not differ by gender.

**Figure S2. Histograms of worry, age and hunger levels**

**A.** Histogram of worry frequency by gender (green – girls, purple – boys); the y-axis tracks percentage. Approximately, 30% of girls and 40% of boys reported never worrying. **B.** Histogram of age of surveyed participants. **C.** Histogram of reported hunger frequency by gender. About half of the sample, across both boys and girls, reported never experiencing hunger.

**Figure S3: Results of worry multiverse by sample selection**

Estimation plot visualizing the estimates for the beta coefficient per sample selection decision. Each dot is a single estimate. Black lines denote the standard deviation of the distribution and white breaks – the mean. The bottom plot visualizes the permutation-based mean difference between the estimates from each subsample and all data. Distributions that do not cross the black line (0) suggest statistically reliable differences between the estimate of the relevant subsample (e.g. Western Pacific Region (WPR), South-East Region (SEAR) and Americas) and the estimate from the full sample. We have coded countries into regions using the WHO regions classification.

**Figure S4: Results of worry multiverse by predictor preprocessing**

**Figure S5: Results of worry multiverse by outcome preprocessing**

**Figure S6: Results of worry multiverse by covariate selection.**

**Figure S7: Results of worry multiverse by random structure**

Note that for computational tractability, we randomly sampled 1000 models per group.

**Figure S8: Results of worry multiverse by sampling weights**

Note that for computational tractability, we randomly sampled 1000 models per group.

**Figure S9: Results of suicidality multiverse by sample selection**

**Figure S10: Results of suicidality multiverse by predictor preprocessing**

**Figure S11: Results of suicidality multiverse by outcome preprocessing**

**Figure S12: Results of suicidality multiverse by covariate selection**

**Figure S13: Results of suicidality multiverse by random structure**

Note that for computational tractability, we randomly sampled 1000 models per group.

**Figure S14: Results of suicidality multiverse by sampling weights**

Note that for computational tractability, we randomly sampled 1000 models per group.

**Figure S15. Results of worry dose-response multiverse by sample selection**

As in main text Fig. 4A, but across sample selection decisions. Results are largely consistent across different subsamples.

**Figure S16. Results of worry dose-response multiverse by covariate selection**

As in main text Fig. 4B, but across covariate selection decisions. Results are largely consistent across different covariate specifications.

**Figure S17. Results of suicidality dose-response multiverse by sample selection**

As in main text Fig. 4B, but across sample selection decisions. Results are largely consistent across different subsamples.

**Figure S18. Results of suicidality dose-response multiverse by covariate selection**

As in main text Fig. 4B, but across covariate selection decisions. Results are largely consistent across different covariate specifications.
